# Supplementary material for: The dynamics of prebiotic take-off: the transfer of functional RNA communities from mineral surfaces to vesicles
Source: Commun Biol. 2025 Mar 23;8:484. doi: 10.1038/s42003-025-07841-2 (PMC11930959; doi:10.1038/s42003-025-07841-2)
Supplement: Supplementary file 1 — Supplemental material [file 42003_2025_7841_MOESM1_ESM.pdf]

The dynamics of prebiotic take-off: the transfer of functional RNA communities from mineral surfaces to vesicles

Supplementary materials

Dániel Vörös[1,2,3], Tamás Czárán[1*], András Szilágyi[1,3], Balázs Könnyű[1,3]

[1] HUN-REN Centre for Ecological Research, Institute of Evolution, Konkoly-Thege M. út 29-33, Budapest, 1121, Hungary
[2] ELTE Eötvös Loránd University, Institute of Biology, Pázmány Péter sétány 1/C, Budapest, 1117, Hungary
[3] Parmenides Foundation, Hindenburgstr. 15, Pöcking, 82343, Germany
*****email: czaran.tamas@ecolres.hu

Supplementary Note 1

We have tested the evolutionary potential of the MCRS and the SCM by initiating the two systems filled with a specific replicator and its complement. The replicator species chosen for this experiment (see Supplementary Fig. 1) was found in a preliminary SCM simulation with different parameters. It was an extreme cis-trans promiscuous sequence with 3 enzymatic activities on each of its strands, all 6 activities different. So, altogether it could catalyse 6 of the 7 necessary enzymatic activities assumed in the simulation that produced it, in which it persisted (with a quasi-species of small mutational modifications) for ~20 000 generations. We created an alternative enzyme mapping consisting of the 6 activities of this specific cis-trans promiscuous replicator. In MCRS, the initial replicator type disappeared, and the replicator types subsequently evolved had characteristically different properties, consisting mainly of trans-promiscuous replicator types, without cis-promiscuity persisting which is considered normal in MCRS simulations (see Supplementary Fig. 2). Surprisingly, in the SCM simulation the initial hyper-promiscuous type also disappeared almost instantly, and the proportions of other promiscuous types returned to ordinary levels (Supplementary Fig. 3), suggesting that the hyper-promiscuous species was an outlier even in the SCM. These experiments led us to conclude that the low level of cis-promiscuous replicators in the MCRS and the SCM is not due to the lack of the necessary mutants or the limited potential of the system to select for them: they are simply not advantageous in the surface-bound context.

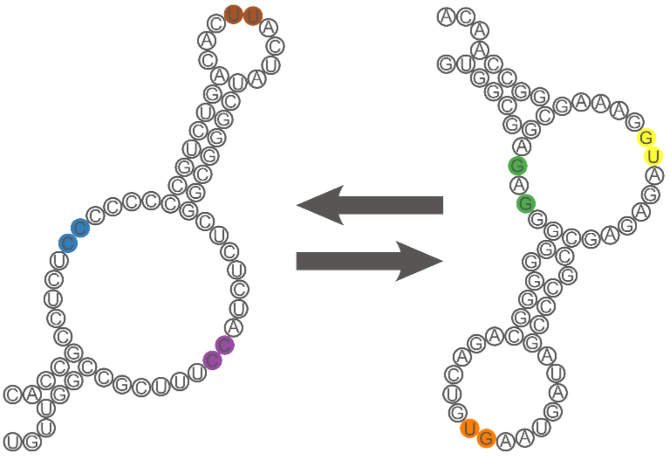

Supplementary Fig. 1. A trans-promiscuous replicator species that is also cis-promiscuous on both of its complementary strands with 3-3 enzymatic activities. It could harbour 6 different enzymatic activities in the SCM simulation that produced it.

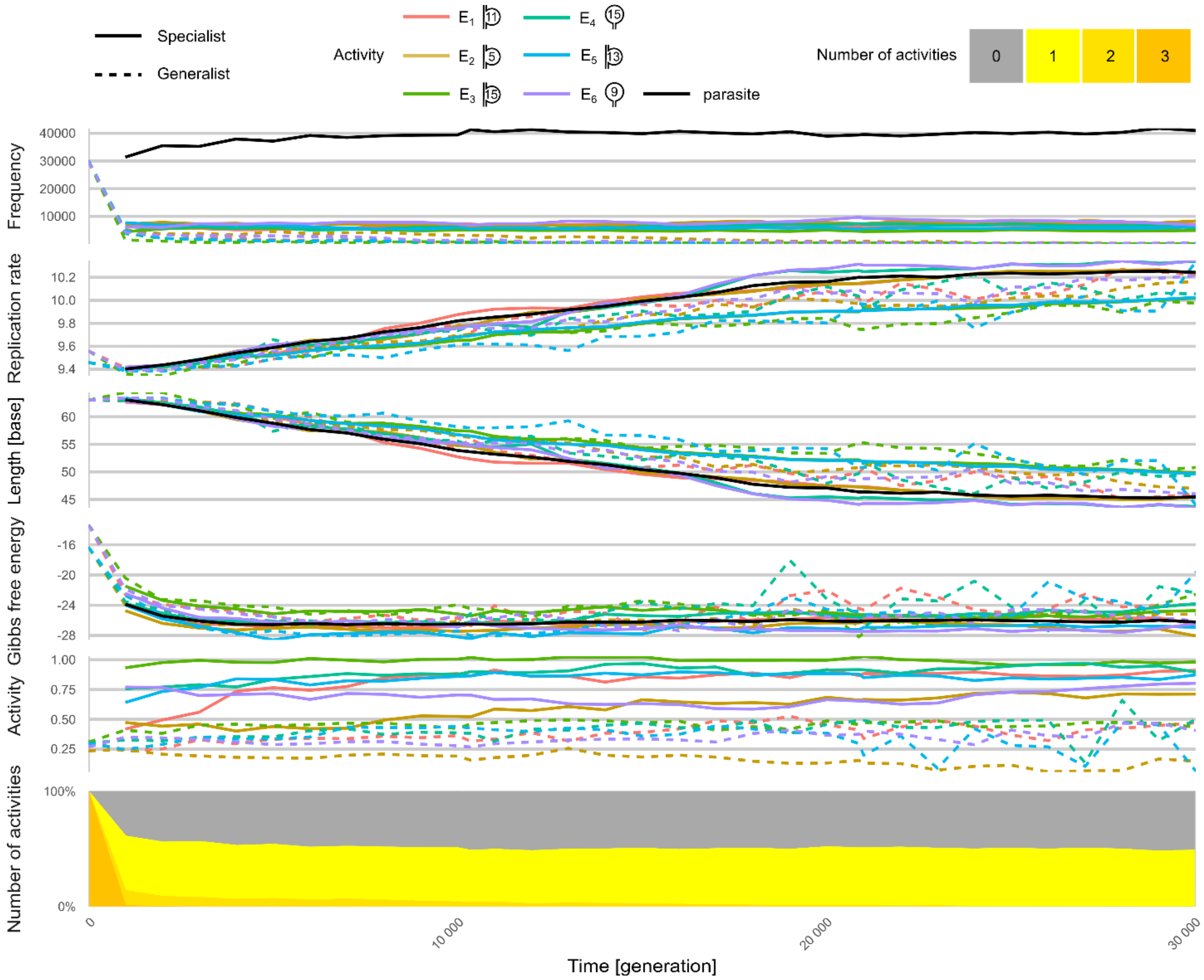

Supplementary Fig. 2. Evolution of traits in an MCRS simulation prepared for the hyper-promiscuous replicator type (Supplementary. Fig. 1). Relevant spatial parameters: $D = 4$, $N_{\text{met}} = 57$, $N_{\text{rep}} = 49$.

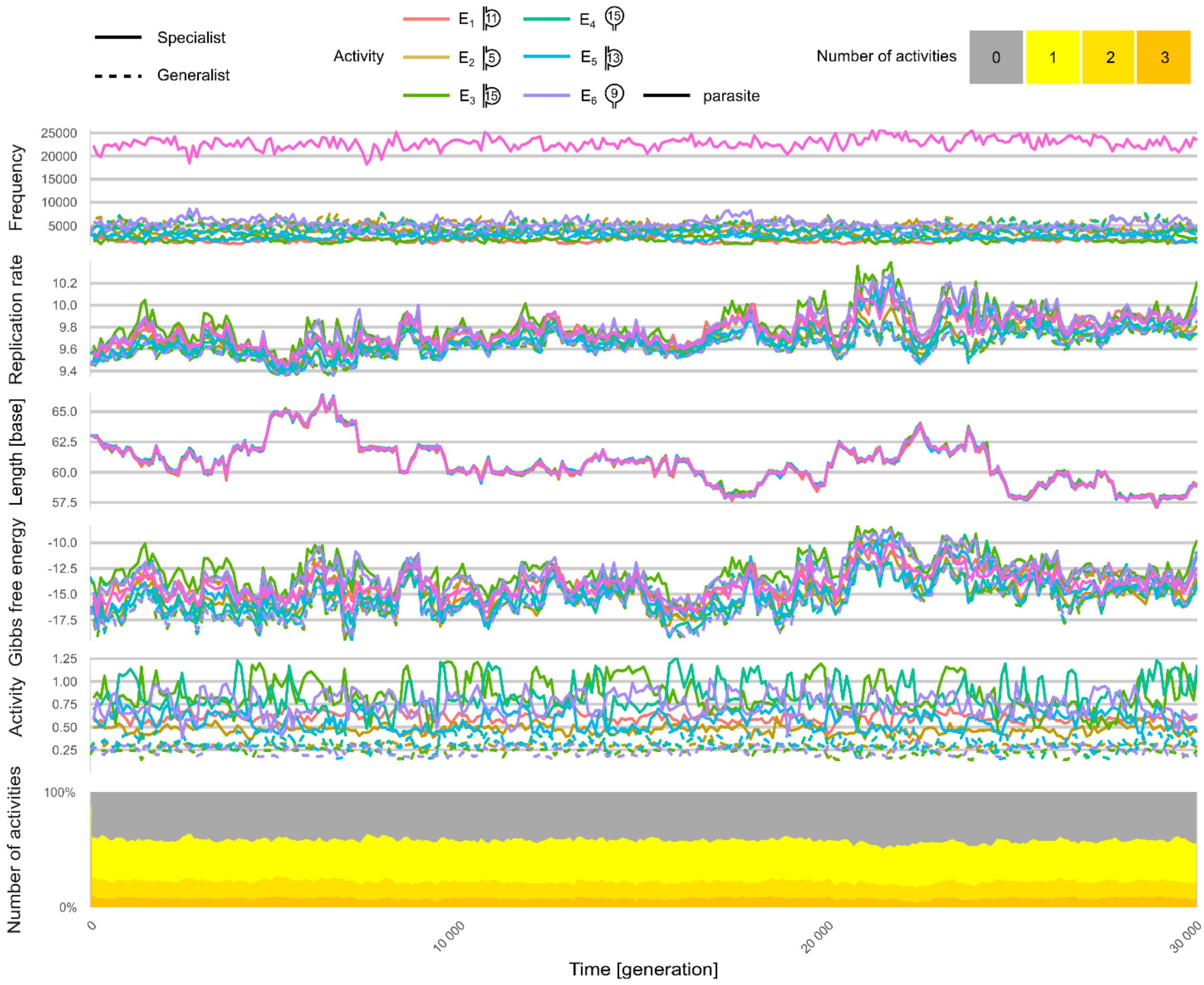

Supplementary Fig. 3. Evolution of traits in an SCM simulation prepared for the hyper-promiscuous replicator type (Supplementary Fig. 1). Relevant spatial parameters: $S = 90$.

Supplementary Note 2

Neighbourhood sizes

The N-sized neighbourhood of a cellular automata model is a sub-lattice consisting of N sites centred on and including the focal site. The smallest neighbourhood is of size N=1, in which the only site included is the focal site itself. On a square lattice as the arena of a cellular automata model, increasing the size of the most isometric neighbourhood yields $N = 5, 9, 13, 21, 25, \ldots$ for sterical reasons. See Supplementary Fig. 4. Notable neighbourhood sizes are the von Neumann neighbourhood ($N = 5$) and the Moore neighbourhood ($N = 9$). Another - not regular - neighbourhood used in the MCRS model is the Toffoli-Margolus neighbourhood, which contains 4 sites with the focal site in the top left corner of a 2x2 sub-lattice.

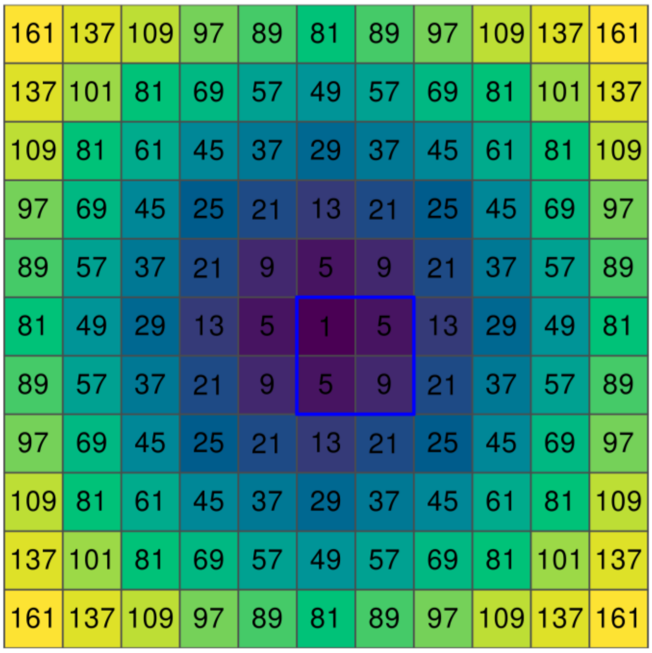

Supplementary Fig. 4. The different neighbourhood sizes. Background colours show the border of neighbourhoods, and the numbers inside squares indicate the number of sites within the neighbourhood (sites with lower numbers included). The focal site is in the centre (1). Blue border shows the Margolus neighbourhood of the focal site.

Supplementary Table 1

The evolving traits of replicators.

Variable	Description
L	number of bases in the replicator sequence
ΔG_{min}	Gibbs free energy of the dominant secondary structure
P_{fold}	probability of being in the dominant folded state
P_{deg}	probability of replicator degradation during a time step
R	Replicability
m	number of active sites in a sequence
a	strength of enzymatic activity
