# Supplementary material for: The dynamics of prebiotic take-off: the transfer of functional RNA communities from mineral surfaces to vesicles
Source: Commun Biol. 2025 Mar 23;8:484. doi: 10.1038/s42003-025-07841-2 (PMC11930959; doi:10.1038/s42003-025-07841-2)
Supplement: Supplementary file 2 — Reporting summary [file 42003_2025_7841_MOESM2_ESM.pdf]

nature portfolio

Corresponding author(s): Tamás Czárán
Last updated by author(s): Nov 21, 2024

Reporting Summary

Nature Portfolio wishes to improve the reproducibility of the work that we publish. This form provides structure for consistency and transparency in reporting. For further information on Nature Portfolio policies, see our Editorial Policies and the Editorial Policy Checklist.

Statistics

For all statistical analyses, confirm that the following items are present in the figure legend, table legend, main text, or Methods section.

n/a	Confirmed	
☐	☒	The exact sample size (*n*) for each experimental group/condition, given as a discrete number and unit of measurement
☐	☒	A statement on whether measurements were taken from distinct samples or whether the same sample was measured repeatedly
☒	☐	The statistical test(s) used AND whether they are one- or two-sided<br>*Only common tests should be described solely by name; describe more complex techniques in the Methods section.*
☒	☐	A description of all covariates tested
☒	☐	A description of any assumptions or corrections, such as tests of normality and adjustment for multiple comparisons
☐	☒	A full description of the statistical parameters including central tendency (e.g. means) or other basic estimates (e.g. regression coefficient) AND variation (e.g. standard deviation) or associated estimates of uncertainty (e.g. confidence intervals)
☒	☐	For null hypothesis testing, the test statistic (e.g. *F*, *t*, *r*) with confidence intervals, effect sizes, degrees of freedom and *P* value noted<br>*Give P values as exact values whenever suitable.*
☒	☐	For Bayesian analysis, information on the choice of priors and Markov chain Monte Carlo settings
☐	☒	For hierarchical and complex designs, identification of the appropriate level for tests and full reporting of outcomes
☒	☐	Estimates of effect sizes (e.g. Cohen's *d*, Pearson's *r*), indicating how they were calculated

Our web collection on statistics for biologists contains articles on many of the points above.

Software and code

Policy information about availability of computer code

Data collection	Data was gathered by own custom C++ code. All results can be reproduced with the codes supplied in our GitHub repository https://github.com/danithered/mcrscm. We have used Vienna RNA package (version 2.6.3) to calculated RNA secondary structures.
Data analysis	To analyze the data we have used custom C++ codes and R (version 4.4.1) software with packages dplyr, tidyr and ggplot2. All neccessary custom codes can be found on our GitHub repository https://github.com/danithered/mcrscm.

For manuscripts utilizing custom algorithms or software that are central to the research but not yet described in published literature, software must be made available to editors and reviewers. We strongly encourage code deposition in a community repository (e.g. GitHub). See the Nature Portfolio guidelines for submitting code & software for further information.

Data

Policy information about availability of data

All manuscripts must include a data availability statement. This statement should provide the following information, where applicable:
- Accession codes, unique identifiers, or web links for publicly available datasets
- A description of any restrictions on data availability
- For clinical datasets or third party data, please ensure that the statement adheres to our policy

Our data is generated by simulations. Data corresponding to our main results can be found in our GitHub repository https://github.com/danithered/mcrscm. The rest of the data can be reproduced with our codes we have supplied in the GitHub repository.

Human research participants

Policy information about studies involving human research participants and Sex and Gender in Research.

Reporting on sex and gender	We have not gathered data from human participants.
Population characteristics	*Describe the covariate-relevant population characteristics of the human research participants (e.g. age, genotypic information, past and current diagnosis and treatment categories). If you filled out the behavioural & social sciences study design questions and have nothing to add here, write "See above."*
Recruitment	*Describe how participants were recruited. Outline any potential self-selection bias or other biases that may be present and how these are likely to impact results.*
Ethics oversight	*Identify the organization(s) that approved the study protocol.*

Note that full information on the approval of the study protocol must also be provided in the manuscript.

Field-specific reporting

Please select the one below that is the best fit for your research. If you are not sure, read the appropriate sections before making your selection.

☐ Life sciences ☐ Behavioural & social sciences ☒ Ecological, evolutionary & environmental sciences

For a reference copy of the document with all sections, see nature.com/documents/nr-reporting-summary-flat.pdf

Ecological, evolutionary & environmental sciences study design

All studies must disclose on these points even when the disclosure is negative.

Study description	We have simulated evolutionary scenarios of hypothetical RNA ribozymes in two spatial settings: on mineral surfaces and in vesicles.
Research sample	We have sampled agent-based simulations with approximately 90 000 agents. Each simulations were repeated at least 5 times and run until they reached equilibrium.
Sampling strategy	We have sampled our simulations in each 100th or 1000th generations. We than calculated mean and SD values. No hypothesis testing was required as we had data only from simulations, so exploratory analyses were sufficient.
Data collection	Automatic reporter functions collected the data.
Timing and spatial scale	We have collected data at each 100th or 1000th generations. More frequent data collection was not possible, due to the time and disk space consumption of the sampling.
Data exclusions	We have gathered data in a wide range of parameters, but we have presented only the non-redundant results.
Reproducibility	All our results can be reproduced with the codes supplied in our GitHub repository. Please note, that minimal differences can arise due to the stochastic nature of the simulations.
Randomization	We used stochastic simulations, so the question is not really relevant. For repeated measures, we have used different random seeds.
Blinding	No data was collected by hand, so blinding is not relevant in our study.

Did the study involve field work? ☐ Yes ☒ No

Reporting for specific materials, systems and methods

We require information from authors about some types of materials, experimental systems and methods used in many studies. Here, indicate whether each material, system or method listed is relevant to your study. If you are not sure if a list item applies to your research, read the appropriate section before selecting a response.

Materials & experimental systems

n/a	Involved in the study
☒	☐ Antibodies
☒	☐ Eukaryotic cell lines
☒	☐ Palaeontology and archaeology
☒	☐ Animals and other organisms
☒	☐ Clinical data
☒	☐ Dual use research of concern

Methods

n/a	Involved in the study
☒	☐ ChIP-seq
☒	☐ Flow cytometry
☒	☐ MRI-based neuroimaging
